# Advances in the Bioinformatics Knowledge of mRNA Polyadenylation in Baculovirus Genes

**DOI:** 10.3390/v12121395

**Published:** 2020-12-06

**Authors:** Iván Gabriel Peros, Carolina Susana Cerrudo, Marcela Gabriela Pilloff, Mariano Nicolás Belaich, Mario Enrique Lozano, Pablo Daniel Ghiringhelli

**Affiliations:** 1 Laboratorio de Ingeniería Genética y Biología Celular y Molecular—Área Virosis de Invertebrados, Departamento de Ciencia y Tecnología, Instituto de Microbiología Básica y Aplicada, Universidad Nacional de Quilmes, Roque Sáenz Peña 352, B1876BXD Bernal, Buenos Aires, Argentina; iperos@inibibb-conicet.gov.ar (I.G.P.); mbelaich@unq.edu.ar (M.N.B.); pdg@unq.edu.ar (P.D.G.); 2 Laboratorio de Virus Emergentes, Departamento de Ciencia y Tecnología, Instituto de Microbiología Básica y Aplicada, Universidad Nacional de Quilmes, Roque Sáenz Peña 352, B1876BXD Bernal, Buenos Aires, Argentina; mpilloff@unq.edu.ar (M.G.P.); mario.lozano@unq.edu.ar (M.E.L.)

**Keywords:** *Baculoviridae*, mRNA, polyadenylation process, RNA structure, pattern-searching

## Abstract

Baculoviruses are a group of insect viruses with large circular dsDNA genomes exploited in numerous biotechnological applications, such as the biological control of agricultural pests, the expression of recombinant proteins or the gene delivery of therapeutic sequences in mammals, among others. Their genomes encode between 80 and 200 proteins, of which 38 are shared by all reported species. Thanks to multi-omic studies, there is remarkable information about the baculoviral proteome and the temporality in the virus gene expression. This allows some functional elements of the genome to be very well described, such as promoters and open reading frames. However, less information is available about the transcription termination signals and, consequently, there are still imprecisions about what are the limits of the transcriptional units present in the baculovirus genomes and how is the processing of the 3′ end of viral mRNA. Regarding to this, in this review we provide an update about the characteristics of DNA signals involved in this process and we contribute to their correct prediction through an exhaustive analysis that involves bibliography information, data mining, RNA structure and a comprehensive study of the core gene 3′ ends from 180 baculovirus genomes.

## 1. Introduction

In the recent years, the next-generation sequencing (NGS) technologies produced a marked increase in the availability of genomic sequences, which deep knowledge involves the use of bioinformatic approaches. Genomes must be characterized individually and comparatively to provide a global description that allows extract the stored biological information and to establish the molecular basis for each different phenotype. Therefore, the use of bioinformatics tools to analyze data from high-throughput functional genomic approaches is a relevant strategy to improve the functional genome annotation. One of the first steps in this field involves the correct identification of the sequences that encode proteins (ORFs: open reading frames). This process is often complex in eukaryotic genomes, due to mRNA splicing, poor knowledge about regulatory sequences (e.g., promoters) and because the protein-coding genomic fraction is usually a minority. Different de novo gene prediction algorithms use hidden Markov models (HMM) or other statistical methods to recognize ORFs, start and stop codons, polyadenylation signals, promoter sequences, and other characteristics that are indicative of coding regions [1]. However, the de novo gene discovery is partially dependent on the organism analyzed, since compositional differences such as GC content and codon frequency generate biases that must be considered. Although there are algorithms that recognize these characteristics for some species when a large number of coding sequences are available [2,3,4], currently there is none specialized for many other biological entities including viruses. In particular, this lack of tools and information exists for *Baculoviridae*, a viral family with broad biotechnological uses.

In order to develop a useful algorithm about this issue it is firstly necessary to determine the characteristics of the sequence set under study. mRNA polyadenylation is closely linked to transcription termination and is also involved in the transport of transcripts to the cytoplasm, in the process of protein translation and mRNA decay [5,6]. Therefore, a deep analysis on this topic will allow better genomic annotations and a greater knowledge about the genetics of the entity under observation. Regarding to this, the knowledge about the mRNA polyadenylation from protein-coding genes in *Baculoviridae* is fragmented and requires its integration and enhancement.

In this review, we provide an update about the characteristics of DNA signals involved in the baculovirus polyadenylation process and their prediction using a selected set of 180 complete genomes stored in GenBank, bibliographic data mining, standard bioinformatics tools and pipelines ad hoc designed.

## 2. Actual Genomic Knowledge about the Family *Baculoviridae*

**Figure 1:**
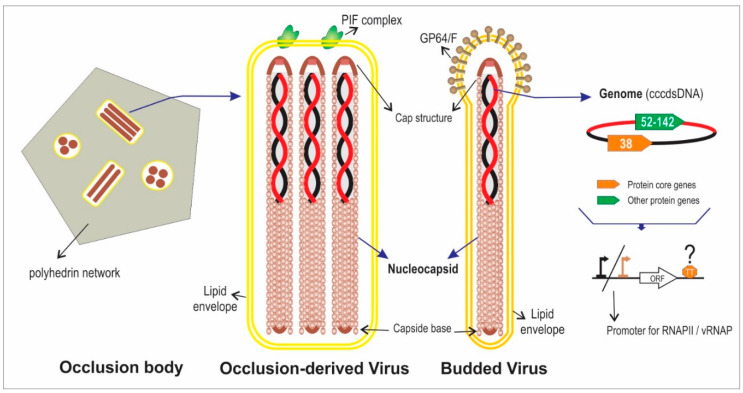
Baculovirus infective phenotypes. The illustration represents the typical characteristics of baculoviruses. The occlusion body (OB) for betabaculoviruses (not illustrated), is granular in shape (compose of a granulin network) and usually contains 1 occlusion-derived virus (ODV) with 1 nucleocapsid. In the other genera, OBs have a polyhedral shape (as illustrated in the figure) and ODVs can be “multiple” (containing several nucleocapsids as represented in the illustration) or “single” (containing 1 nucleocapsid). The multiprotein complex named as PIF (Per os Infectivity Factor) is responsible for the primary infection in the host. The Budded viruses (BV) contain 1 nucleocapsid. The fusogenic proteins GP64 (Group I alphabaculoviruses) or F (remaining baculoviruses) mediate the entry of the BVs into the larval cells (secondary infection). The lipid envelopes of both virions (ODV and BVs) have different composition. The genome is a cccdsDNA of 80–180 kbp containing 100–200 protein genes (38 of which are shared by all baculoviral species). RNAP II: RNA polymerase II (from host); vRNAP: viral RNA polymerase (encoded by baculoviruses); TT: Transcription terminator. This review focuses on the 3′ end of baculoviral protein genes.

*Baculoviridae* is a viral family which members are infective for insects (larval stage) and are characterized by possessing a covalently closed circular double strand DNA (cccdsDNA) genome, with sizes between 88 and 200 kbp, and encoding for 90 to 180 proteins. Some of these viruses are used as bioinsecticides for the control of agricultural pests [7,8,9]. Additionally, these viruses are used as foreign protein expression systems in vitro or in vivo and as delivery systems for therapeutic genes in mammalian cells [10,11]. Baculoviruses have been isolated from different orders of insects (Lepidoptera, Hymenoptera and Diptera) and have two infective morphologies: budded virus (BV) and occlusion derived virus (ODV) [12] (Figure 1). BVs appear early in infected cells, spreading the infection throughout the body of the larva and are composed of a nucleocapsid (viral DNA and different associated proteins), enveloped by a membrane derived from the infected cells. By contrast, ODVs are produced in the late stage of the infection and the enveloped nucleocapsids (one or more according to species) are embedded in an occlusion body (OB), that are constituted by a protein matrix formed by polyhedrin or granulin according to genus.

Based on sequence information, OB morphology and susceptible hosts, *Baculoviridae* is divided into 4 genera: *Alpha*-, *Beta*-, *Gamma*- and *Deltabaculovirus*. The first two include viruses that infect lepidoptera, while the other two contain pathogens for hymenoptera and diptera, respectively [13,14,15]. The alphabaculoviruses are further subdivided into Group I and Group II, depending on the fusion protein they encode and other gene content: Group I express the GP64 fusion protein, while Group II uses the F protein [15,16].

There are a large number of experimental works published, mainly referred to particular genes, genomic regions or complete genomes, but only few reports have shown global comparative analyzes based on all available genomes. In this sense, there are studies focused on the determination of gene orthology or genome evolution [17,18,19,20], where it was determined that there are at least 38 core genes (protein encoding sequences shared by all members of the family). The other baculoviral genes are typical of genus, of small subsets or unique per species [21]. Additionally, phylogenetic analysis based on multiple alignment of concatenated baculovirus core protein sequences confirmed the current classification in four genera and showed that both *Alpha*- and *Betabaculovirus* have a consistent grouping into two different clades: a and b [7,20].

## 3. Eukaryotic mRNA 3′ End Processing

The eukaryotic pre-mRNA molecules synthesized by the transcription event, goes through three processing steps before being exported as a functional mature messenger: capping; polyadenylation; and splicing. Since there is evidence of homology in polyadenylation signals and proteins involved (e.g., 3′ end processing of yeasts and mammals [22]), it is possible to assume that the mechanism is essentially conserved regardless of small differences in some organisms. Additionally, polyadenylation participates in regulatory mechanisms of gene expression since it produces a great variability of possible phenotypes in response to extracellular and intracellular conditions. In relation to this, it is associated with several important biological mechanisms: the termination of transcription, since the cleavage that occurs in the pre-mRNA destabilizes its interaction with the RNA polymerase II (RNAP II); the intracellular transport of the mRNA to the cytoplasm; and its half-life. It also ensures that the transcript translation is correct, tags the pre-mRNA that will continue the maturation stages, and delimits the last intron to be excised during the splicing process [23].

**Figure 2:**
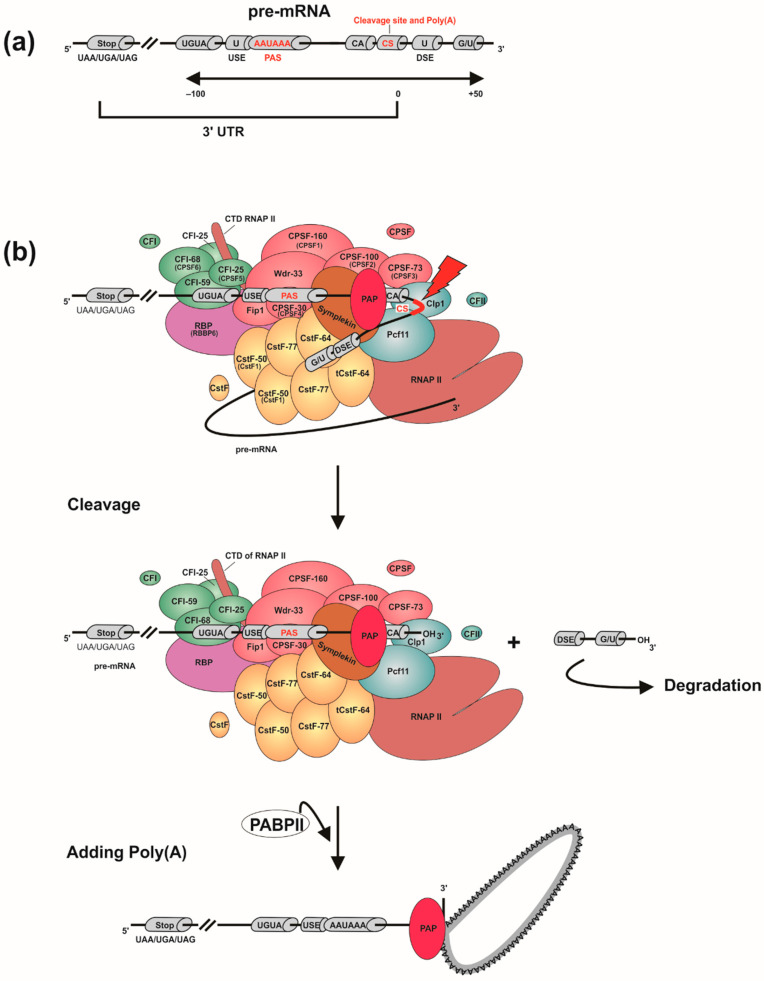
Eukaryotic polyadenylation process. DNA sequence elements involved in polyadenylation (**a**); and the polyadenylation mechanism (**b**) in mammalian pre-mRNA. The different protein factors involved in the process are identified by their names most frequently used in the literature. PAS: *Polyadenylation signal*; USE: *Upstream sequence element*; DSE: *Downstream sequence element*; CS: *Cleavage site*; CPSF: Cleavage and polyadenylation specific factor; CstF: Cleavage stimulation factor; CFI and CFII: Cleavage factors I and II; RNAP II: RNA polymerase II; PAP: Polyadenylate polymerase; RBP: Retinoblastoma-binding protein 6; PABPII: Poly(A) binding protein II.

Particularly, the polyadenylation process begins during transcription when the cleavage and polyadenylation specific factor (CPSF) [24], associated with the RNAP II carboxyterminal domain (CTD), recognizes in the pre-mRNA a polyadenylation signal (PAS) or positioning element (PE), which has the “AAUAAA” consensus sequence (Figure 2a). In the proximity of the PAS are the upstream sequence element (USE) and the downstream sequence element (DSE). The USE has U-rich sequences and is located 40–100 nt upstream from the polyadenylation site [25]. The DSE has U- and GU-rich sequences and is located 10–70 nt downstream from the polyadenylation site. These two elements facilitate the PAS recognition while their relative positions and sequence variations mediate the strength of the polyadenylation signal. In this way, they form the USE-PAS-DSE signaling pattern, which is recognized by the CPSF and is highly conserved in eukaryotes [26]. Eventually, “UGUA” motifs upstream to the PAS and an auxiliary downstream sequence element (Aux-DSE) to the PAS may also appear [27,28]. The Aux-DSE has G-rich sequences and works by interacting with regulatory factors and stimulating 3′ end processing of the pre-mRNA.

When the CPSF detects the PAS (Figure 2b), the cleavage stimulation factor (CstF) [29], which recognizes the DSE, associates with the CTD of the RNAP II and together with the CPSF recruit two cleavage factors (CFIm, which recognizes USE, and CFIIm), Symplekin and CPSF3 endonuclease, in order to form the cleavage complex. This multiprotein complex cuts the pre-mRNA at the cleavage site (CS), located 10–30 nt downstream to the PAS and 0–20 nt upstream to the DSE (usually after a “CA” dinucleotide), leaving a free 3′ hydroxyl [26]. Consequently, the CPSF is released from the transcript but the RNAP II continues to elongate the sequence until it reaches a Transcription end site (TES). Next, the polyadenylate polymerase (PAP) adds up to 250 adenosines at the 3′ end of the pre-mRNA, giving stability to the polyadenylated transcript. The poly(A) binding protein (PABP) is the factor responsible for controlling the length of the poly(A) tail, as it mediates the interaction between CPSF and PAP [30]. The length of the poly(A) tail is variable among species belonging to different eukaryotic kingdoms. In humans, for example, it has an extension of 250–300 residues, while in yeasts only 70–80 adenines are added [5]. Then, when the pre-mRNA is transported to the cytoplasm, part of the poly(A) tail is cleaved leaving between 30–50 adenines. Pre-mRNAs that were not polyadenylated are degraded by cytoplasmic processing-bodies (P-bodies) [31], while those with the poly(A) tail can continue to be processed until they become mature mRNA and subsequently translated into polypeptides. Moreover, it has been discovered that by mutating the PAS, the polyadenylation process is blocked and the TES is interrupted, causing the RNAP II to continue elongating the transcript in the *3′* untranslated region (3′-UTR) [32]. In addition, the advances in massively parallel sequencing techniques (e.g., RNA-Seq) allowed that the presence of multiple polyadenylation sites in pre-mRNAs (which generates different isoforms) had been revealed in more than 70% of human genes [5]. These findings show the existence of an Alternative polyadenylation (APA) process (review in [6]).

Besides, it is important to mention that the polyadenylation mechanism is regulated through different ways: the correct positioning of the sequence elements present in the 3′ end, and their nucleotide composition [6,33] and structural environment [34,35,36,37,38]; the recruitment of polyadenylation factors in promoter regions [33,39] and their concentration [40,41,42]; the RNAP II elongation speed [43]; the presence of structured regions (Aux-DSEs, local chromatin conformation, presence of nucleosomes and epigenetic marks) [6,44]; the concentration of transcriptional elongation factors [6,45,46]; the existence of RNA binding proteins [33]; the presence of splicing factors [47,48,49,50,51]; and the activation of specific signaling pathways [52,53].

## 4. How Bioinformatics Can Assist in the Characterization of the Baculoviral mRNA 3′ End Processing

In *Baculoviridae*, hundreds of protein genes have been reported, but many of them are still under “hypothetical” status [12,17]. Generally, the presence of ORFs of a certain length (e.g., minimum of 50 amino acids) and little or no overlap between neighbors are usually the determining elements to postulate the existence of protein genes. However, a more complex analysis that includes promoter motifs and transcription termination signals would make it possible to improve gene prediction. In this sense, a comprehensive bioinformatic study of the 3′ end processing mechanism of baculoviral genes will allow to generate significant knowledge about which are the main sequence elements involved, how this process occurs and how it is regulated in infected insect cells and it will assist, for example, to reinforce the status of many hypothetical genes. Previous reports support that not only the factors that make up the polyadenylosome are critical for the development of this mechanism in *Baculoviridae*, but also the presence of cis-acting sequence elements and their positions, together with secondary structures adopted by the baculoviral pre-mRNAs, arranged in specific regions of the 3′ end, could participate in the regulation of the interaction with the cellular transcriptional machinery. Consequently, it is important to analyze and characterize comprehensively, the composition and structure of mRNAs ends and downstream regulatory regions of the baculoviral genes using different in silico approaches.

### 4.1. Bioinformatic Detection of Signals Involved in the Processing of the 3′ End of Genes

The identification of PASes by the use of in vitro techniques has contributed to the characterization of the pre-mRNA polyadenylation process in different organisms. Comparative genomic and transcriptomic studies enable the analysis of the molecular basis, the regulation, and the biological implications of this complex mechanism.

DNA microarrays have been used to analyze the processing of the 3′ end of genes in different organisms and to determine the relative use of distal versus proximal PASes. In these studies, an extended probe was used to visualize the pre-mRNA isoforms generated by the processing of distal PASes, and a common probe to determine the isoforms obtained as products of polyadenylation in all PASes (both proximal and distal) [5,54]. This technique also allowed to show the relationship between the processing of the 3′ end of genes and cell proliferation, since in this biological process a higher signal was obtained from the common probe, associated with the shortening of the 3′-UTR by the use of proximal PASes during polyadenylation [55]. However, the microarray technique has some disadvantages in terms of the limited design of the probes, such as: the partial hybridization that can occur over the total genes; the dependence with already characterized PASes, since it does not allow identifying new motifs; and the quantification of mRNA with multiple polyadenylation sites [5].

Some of the newest techniques for the detection of polyadenylation events include polyadenylation site sequencing (PAS-Seq) [56], APA site sequencing (SAPAS) [57], poly(A) site profile determination by sequencing (3P-Seq) [58], deep sequencing of the 3′ end (3′ RNA-Seq) [59], and the extraction of the 3′ region and its deep sequencing (3′ READS) [60]. In different investigations that have used some of the mentioned methodologies, it has been concluded that polyadenylation is a phenomenon that occurs in all eukaryotic pre-mRNAs analyzed (including yeasts, plants, insects, vertebrates, mammals and humans), capable of regulating gene expression and produce protein diversity. Regarding to this, the number of PASes identified and characterized in different organisms has been increased, also detecting them in long non-coding RNAs (lncRNA). Moreover, it has favored the study of the effect of changes in the relative levels and sequences of proximal and distal PASes that influence the polyadenylation event [5]. On the other hand, the study by Hoque and collaborators [60] has used the 3′ READS technique as an alternative option to avoid oligonucleotide (dT) complementarity problems in polyadenylation signals, with the aim of mapping the presence of PASes in the complete mouse genome. As a result of the investigation, 5000 previously described PASes have been found, and it has been determined that 79% of the coding mRNAs and 66% of the non-coding RNAs were processed at their 3′ end through events of APA, although their PAS-usage profiles in introns and upstream exons were different [60].

Given that these experimental methodologies to detect PASes are complex to carry out, the use of bioinformatics tools have emerged as an excellent option to complement them (Table 1). An in silico approach for mapping polyadenylation events, without resorting to gene-by-gene sequencing, may be based on searching in specialized databases such as expressed sequence tag (EST) [61], containing 3′ end sequences of different mRNAs. This option made it possible to obtain information that generated great advances in the knowledge of polyadenylation mechanisms including, for example: 54% of human transcripts are alternately polyadenylated, while in mice APA events occur in 32% of their pre-mRNA [62]; PASes have a general location at 50 nt upstream to poly(A) sites and 15–20 nt upstream to CS; the “AAUAAA” canonical motif was identified in approximately 60% of the polyadenylation sites and nine other non-canonical PASes were detected in 14% of the poly(A) sites in human and murine transcripts (in addition to other upstream and downstream auxiliary signals such as the “UGUA” motif, recognized by the CFIm and located 40–100 nt from the CS) [63]; and in pre-mRNAs with multiple poly(A) sites, the proximal PASes are usually weak and have non-canonical sequences, while those located in the distal zone of the 3′-UTR employ the canonical sequence, making them the strongest signals [5]. Besides, Multiple sequence alignment (MSA) with software such as *Clustal* [64]*,* Muscle [65] among others, can be used to detect PASes. In the investigation by López-Camarillo and collaborators [66], this approach has been used to perform a complete analysis of the 3′ gene ends of *Entamoeba histolytica*. This study has allowed detecting protozoan sequences involved in the processing of pre-mRNA and comparing them with those identified in yeast and human transcripts, to determine similarities and differences, in order to trace the evolutionary distance among these species. Another approach is the clustering or grouping of putative functional signals according to different criteria. For example, cis-acting elements implicated in the human pre-mRNA polyadenylation have been determined by their classification regarding to the strength of the PASes and other nearby auxiliary motifs [67].

Furthermore, an interesting alternative is to use probabilistic prediction based on HMM’s application in biological sequence analysis [86], which involves a statistical modeling of biological sequences to determine the evolution of observable events that depend on internal not-observable-factors. This method allowed to characterize the signals involved in polyadenylation events in the cytoplasm, where cytoplasmic polyadenylation element binding proteins (CPEBs) interact [87]. A strategy linked to HMM involves the determination of conserved motifs. This can be done using programs that detect patterns in a set of sequences (aligned or not) such as MEME SUITE [74] or *RSAT* server [88], or by building sequence logos (graphical representation of the information contained in the MSA) with WebLogo [76] and detecting conserved regions. This approach has allowed locating signals and binding sites for different factors involved in polyadenylation in the human transcriptome [89]. Moreover, there are several algorithms and programs for bioinformatic prediction of the RNA secondary structure, since the characterization of the 3′-UTR structures is an important factor in identification of PASes, as will be detailed in the next section. Furthermore, various bioinformatic applications have been developed, with the specific objective of identifying PASes in different organisms using statistical criteria for the selection and discrimination of these motifs. Some of them predict PASes only in specific organisms while others are more general; in turn, some use particular algorithms while others are based on a combination of several algorithms mentioned above.

In summary, in silico studies allow the search of PASes in target RNAs of defined species, in order to characterize genomic contexts, to phylogenetically compare them within the same genus and to investigate how they are regulated by other sequence elements and other accessory proteins. The ultimate goal of such analysis is to exhaustively characterize the polyadenylation mechanism and understand its association with other biological processes including the manifestation of phenotypes, which occur in response to the environmental stimuli to which the organism under study is exposed.

### 4.2. Bioinformatic Prediction of RNA Structure

RNA is an essential biological macromolecule for all organism, since it encodes genetic information, regulates gene expression and catalyzes cellular reactions important for its function [90]. It can form many secondary and tertiary structures by means of intramolecular interactions, which mainly occur among the ribonucleotide bases of primary sequence. These structures arise from the spatial arrangement adopted by RNA when it is modulated by different factors: the physicochemical variables of the intracellular environment (temperature, pH, ionic charge, presence of metal ions and metabolites, among others); the genomic context (mutations and modifications of the native RNA); and the interactions with other biomolecules (RNA-proteins, RNA-DNA and intra- and intermolecular RNA-RNA) [91].

These structures are essential for the functional capacity, processing, and stability of RNA, as well as for the development of various genetic phenomena in which RNA participates. Among the most important can be mentioned: polyadenylation; alternative splicing; protein synthesis; cap independent translation mediated by the internal ribosome entry site (IRES) [92]; chromatin remodeling; cellular signals regulation [93]; subcellular location of transcripts; RNA binding proteins association; RNA intermolecular interactions; microRNAs stabilization and regulation [94,95].

**Figure 3:**
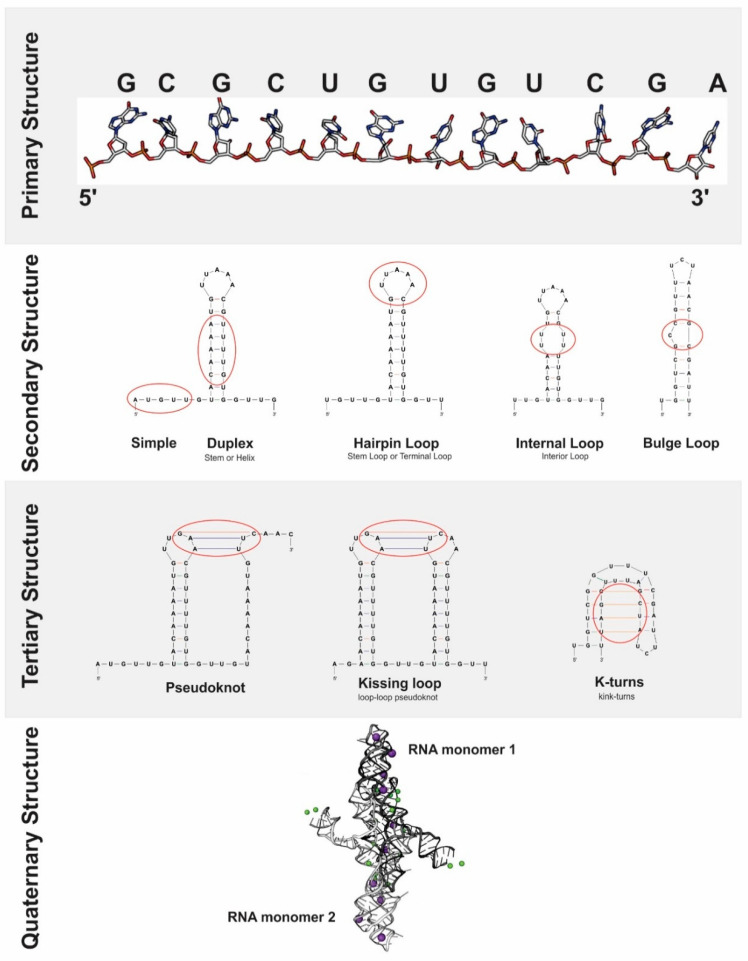
RNA Structures. RNA is a very important molecule for many processes within the cell and its activity is largely determined by its structure (the way it is folded on itself). Although in most cases RNA is a single-stranded molecule, the most stable conformation of a nucleic acid is double-stranded, which is why RNA molecules tend to adopt secondary and tertiary structures by means of intramolecular interactions among the ribonucleotide bases of primary sequence, and even quaternary structures. The illustrated examples are the common structures that RNA molecules adopt in cells.

Spatially, RNA tends to form a duplex (stem or helix) due to the nucleotide base pairing at different points of the molecule, generating several secondary structures (Figure 3): stem loops; internal loops; and bulge loops; among others [96]. Tertiary RNA structures are determined from the conformation adopted by the interactions among stable secondary structure units, which are stabilized by the stacking of nearby duplexes, cations that regulate electrostatic forces and structure-shaping proteins [97] avoiding the formation of stable alternative secondary structures that prevent tertiary structures assembly. In this sense, it is possible to distinguish the functional folds of RNA from non-functional structures, since the former can be validated by physical, enzymatic and chemical surveys, sequencing and phylogenetic analysis, while the latter cannot [91]. Moreover, tertiary structures are also determined by intramolecular interactions, both sequence-specific interactions (in triple-stranded RNAs and receptor-tetra-duplex motifs) and sequence- non-specific (interactions within the molecular skeleton and stacking maximization forces) [97]. Within the set of tertiary structures (Figure 3), there are pseudoknots, kissing loops or loop-loop pseudoknots, Kink-turns, receptor-tetraduplex motifs, T-loops, RNA triplex and G-quadruplexs.

A deep analysis about the function and importance of RNA molecules in the biological processes requires the structural studies under specific intra- and extracellular conditions. Furthermore, the results of these investigations are better if they come from the combination of experimental techniques and computational inferences.

The in vitro study of the functional structure of RNA has been developed from physical techniques and enzymatic assays, although its use for structural modeling is limited [92]. Nuclear magnetic resonance imaging, X-ray crystallography and cryoelectronic microscopy allow characterization of RNA structures and complexes with high resolution, but are limited to in vitro analysis due to the low concentration, length and dynamism of the molecule within the cell [91]. Additionally, by means of enzymatic assays based on the use of specific single or double stranded ribonucleases, it is possible to analyze the accessibility and nucleotide-pairing capacity associated with secondary structure formation, as well as determine RNA-protein binding sites (footprint). However, enzymatic digestions and subsequent analysis of the generated fragments have a lower sensitivity than chemical reagents, since they do not detect small variations in the sequences (such as mismatch) and are limited to in vitro assays. This is due to the inability of the RNAses to penetrate the cellular membrane and the high concentrations of cofactors required [96]. Accordingly, it has been experimentally determined that the in vivo conformation of RNA varies considerably with respect to the structure proposed by in vitro techniques, due to the effect of the different intracellular conditions [38].

Furthermore, the in vivo study of the functional structure of RNA is possible thanks to chemical tests using small molecular probes (<500 Da), capable of entering the cell and detecting, with a molecular resolution, specific nucleotide pairings, structural RNA motifs and protein binding sites [91]. A wide variety of probes have been developed. Some recognize specific nitrogen bases (DMS, CMCT, Ketoxal), and others recognize the hydroxyl radical or acylation of the 2′-hydroxyl groups of the ribose skeleton (NMIA, 1M7, NAI, BzCN), which are then analyzed by primer extension or SHAPE [98,99]. The disadvantage in the use of these probes is that they can interfere with the stability of the RNA-protein and RNA-DNA complexes and could generate inaccurate results [91].

Generally, to carry out the structural determination, the results of the enzymatic and chemical methods are reported from a scale of reactivity corresponding to the probes used. The values obtained are statistically transformed into structural constraints of the RNA molecule, considering background noise, local signal bias and in vivo RNA-protein and RNA-RNA interactions [100]. These experimental data are the origin of the in-silico prediction of the RNA secondary structure, which is the objective of several bioinformatic algorithms called “single sequence”. These are based on thermodynamic principles such as, for example, the principle of minimum free energy (MFE) or that of maximum expected accuracy (MEA) [90]. Moreover, sequence comparison algorithms with a certain degree of homology can be used to formulate possible structures. Having this evolutionary information, these programs are more accurate in prediction than those of Single sequence [101]. MFE-based algorithms assign a ΔG^0^ (Gibbs energy value) for each pair of paired nucleotides based on neighboring residues (Turner’s rule), or a ΔG^0^ associated with missing nucleotides based on size, to calculate the probability of mating using a partition function [90]. From these parameters, the different possible structures for the same RNA sequence are modeled. Thus, the most likely conformation is that which has the lowest total Gibbs free energy, while the rest represent the “suboptimal” structures. However, the prediction of the structure is limited to being a theoretical model because it considers a finite amount of thermodynamic parameters since it is impossible to study the molecule in the cellular context, where it interacts with many variables that cannot be measured [96]. That is why the in silico prediction sometimes departs from the real functional conformation that occurs in vivo. Some of the bioinformatics programs that use MFE as a parameter to predict the RNA secondary structure are Mfold [77], RNAstructure [102] and RNAfold [103]. These programs use algorithms based on window comparison methods, in which the distance between paired residues is limited to a window of a certain length, in order to avoid a loss of precision in the generated prediction (which increases as the sequence is longer). However, this strategy has some disadvantages, since it excludes long-distance mating. So, the spectrum of probable secondary structures is minimized, added to the fact that interactions between residues with a ΔG^0^ lower than an established threshold value are ruled out [90]. MEA-based algorithms such as CONTRAfold [104], CentroidFold [105] and IPknot [106], propose from an RNA sequence the secondary structure that maximizes the expected base pairing accuracy [96]. Finally, the RNAalifold algorithm [103], based on sequence comparison and co-variation analysis, allows to identify similar patterns of sequence variation from an alignment of ortholog sequences. In addition, for the prediction of the RNA structure of interest, it considers that those functionally analogous molecules will have similar structures, even if their sequences are not [91]. Although it is estimated that these algorithms have an accuracy in the prediction of the structure of approximately 80%, currently all predicted structures have not been validated, since the in vivo conformations of most mRNAs and other coding RNAs remain unknown [94]. Therefore, it is important to extend the investigation of RNA structures to various organisms and different conditions to identify new functional types.

A complete study of the folding and structures adopted by RNA molecules should involve the in silico prediction, the in vitro study of the arrangement of atoms in the molecule and their subsequent in vitro and in vivo characterization. In this way, the interconnection of these analyzes makes it possible to achieve a complete understanding of the complex nature of this biological molecule [96]. Specifically, the use of bioinformatics tools in the structural prediction of target RNAs makes it possible to obtain a computational approximation of the conformation adopted by the molecule, based on different physicochemical variables. This allows identifying the presence of different types of structures, according to the degree of base mating, positional restrictions, and the steric effect on the RNA. Once the structure is defined, it is possible to begin to determine specific characteristics of it, proceed to explain its functional aspects and inquire about its possible association with various biological processes.

In this sense, previous investigations allowed to show that the formation of secondary structures in pre-mRNAs consist of a significant factor that helps regulate the polyadenylation mechanism in various organisms. It has been possible to determine the role of RNA conformation in the process, allowing to locate the main sequence elements involved in the polyadenylation event in their correct functional positions. The first studies that revealed the existence of a connection between the RNA structure and the processing of the 3′ end of genes resulted from the analysis of mammalian virus genomes. For example, it has been identified that the presence of a complex secondary RNA structure in HTLV-1 allows an arrangement of the sequence elements PAS and CS in their functional positions, which is required for the 3′ end processing mechanism to be efficient [107]. Moreover, it was concluded for HIV-1 that the structural context was critical for the recognition of a canonical PAS by the 3′ end processing machinery despite the absence of sequence conservation [108]. According to this result, it was determined that the local RNA structure regulates the association capacity with the factors involved in the processing of the 3′ end and, ultimately, the biological mechanism [109,110]. Additionally, the importance of the pre-mRNA structure in the polyadenylation event of eukaryotic genes, mainly in transcripts of yeasts, plants, humans, and other mammals, has also been demonstrated. For example, studies in murine secretory IgM concluded that a stem structure promoted the recognition of a DSE by the CstF [34], and findings in *Schizosaccharomyces pombe* demonstrated the existence of an association between the thermal stability of the RNA structure and the preferential use of specific PASes [35]. Another research mentioned that the Aux-DSE was a polyadenylation stimulating factor, since it was determined that approximately 30% of 244 human pre-mRNAs analyzed contained G-rich downstream auxiliary elements capable of forming G-quadruplex structures [44]. Moreover, it was verified that the 3′-UTR of the coding gene for the tobacco endochitinase (*Nicotiana tabacum*) formed a secondary structure, in which a proximal and a distal PAS were respectively located in a stem and in a loop; and because of this structural difference, the distal PAS was more easily recognized by the CPSF [36]. Other results of human introns demonstrated that the RNA editing mechanism allows the polyadenylation process to be activated by interrupting secondary duplex structures that inhibit it [37]; and in a metagenomics study of *Arabidopsis thaliana*, it was concluded that the U- and A-rich region located upstream of the CS had a secondary structure, while the A-rich region localized superimposed and downstream of the CS was not structured [38].

Summarizing, the results of these investigations have shown that the pre-mRNAs are usually differentially structured in the regions adjacent to the polyadenylation sites, which evidences the relationship between the secondary structure of the pre-mRNAs and the processing of the 3′ end of genes. It has also been determined that variables such as the stability of the stem structures, the presence of single-chain regions in the loops and the existence of G-quadruplex, regulate the effectiveness of the polyadenylation mechanism, by mediating the positioning and binding of the processing factors in their functional locations within the 3′ end.

## 5. Baculoviral mRNA 3′ End Processing

The ends of the baculovirus pre-mRNA molecules are processed to generate functional messengers. Thus, numerous investigations have sought to characterize the polyadenylation process of this viral family and determine if it has similarities with the eukaryotic pre-mRNA processing. First, the question was whether the baculoviral genes were transcribed and processed by their own machinery or by host proteins (or a combination of them) since it is a DNA virus with nuclear replication. To try to answer this question, we will first analyze the results of the experimental published works and then we will contribute with the knowledge provided by bioinformatic analyzes carried out on genomic sequences.

### 5.1. Bibliographic Data Mining

Regarding to the transcription of baculoviral genes, it has been discovered that it is temporarily regulated through the different stages of the infection. Consequently, baculoviral genes can be divided into three classes according to their time of expression: early; late; and undefined (without clear signals that allow assigning them to any of the other classes) genes [12]. It has been identified that host RNAP II recognizes early promoters and is responsible for the transcription of early mRNAs, while viral late promoters are recognized by a viral RNA polymerase (vRNAP). The vRNAP is a protein complex composed of LEF-4 (involved in the capping of the 5′ end), LEF-8 and LEF-9 (both form the catalytic domain, with homologous motifs to prokaryotic and eukaryotic RNAPs), and p47 [111]. The proteins that form the viral transcriptional complex are produced late in the infection cycle; therefore, early transcripts can only be generated by the host’s transcriptional machinery. Additionally, early transcripts start at a common eukaryotic tetranucleotide (“CATG”), while late transcripts start at a different tetranucleotide sequence (“TAAG”). Until now, there are no conclusive reports that determine which machinery transcribes the genes of the undefined category.

In order to determine how are processed the 3′ end of baculoviral transcripts, various works have been reported focused on determining the main gene sequence elements involved in polyadenylation and the putative components of the processing machinery involved. Regarding to this, the *p10* gene of *Autographa californica MNPV* (AcMNPV), which codes for two possible transcripts (one of 2500 nt long and the other of 750 nt long), has been characterized by 3′ Rapid amplification of cDNA ends (3′ RACE) and sequencing [112]. The small transcript is the most abundant and presents two canonical PAS sequences (“AAUAAA”) in the 3′-UTR region, of which the distal is the most used. Adjacent to this distal signal (at 22–38 nt downstream) a GU-rich motif is located, which is associated with the CstF of the insect cell infected by AcMNPV. However, this GU- or U-rich motif is not present downstream of the first “AAUAAA” signal, so it is not used as PAS. In addition, it has been found that both the canonical signals and the GU motif were conserved in the 3′-UTR of the *p10* gene of other baculoviruses, in which the PAS sequence could be “AAUAAA” or “AUUAAA”, a motif used alternately in lepidoptera [112]. This was later confirmed for many other genes using third-generation sequencing methods [113,114]. In turn, it has been discovered that mutations in the canonical sequence could cause changes in the cutting position and, therefore, in the polyadenylation pattern in the pre-mRNA. This result has been observed when changing the “AAUAAA” motif for “AAGGUA”, causing RNAP to recognize the sequence of the next PAS for polyadenylation of the pre-mRNA [112]. This observation showed that the canonical PAS alone was insufficient for the 3′ end processing, leading to the hypothesis that there should be other auxiliary sequence elements nearby in the pre-mRNAs to complement “AAUAAA”, such as GU- or U-rich regions.

On the other hand, Jin and Guarino [115] proposed that the polyadenylation event of late mRNAs was different from that occurring in the early viral genes and cell messengers, which were transcribed and processed by the host cell machinery. In their study, they found U-rich regions within the ORFs of late baculoviral genes that were transcribed, and demonstrated by directed mutagenesis that the canonical eukaryotic PAS upstream of the DSE and the GU-rich regions were not essential for the termination mediated by the vRNAP. By contrast, the U-rich motif was recognized as a termination signal comparable to the termination of independent rho bacterial transcription [115]. Moreover, in the late core gene *ORF67* (AcMNPV’s *ORF81*) of *Bombyx mori NPV* (BmNPV), a U-rich motif located at 7 nt downstream of the stop codon was detected, which functioned as a transcriptional terminator and PAS [116]. However, the result on the importance of U-rich sequences in the termination of the transcription in baculoviruses did not exclude the possibility that other A-rich motifs or other PASes may be additional elements involved in the termination.

Despite the findings of Jin and Guarino, it was demonstrated that in some baculoviruses the canonical PAS is the main transcription termination signal of late expression genes, allowing the mRNA to be polyadenylated. The late gene lef-9 of BmNPV (AcMNPV’s *ORF62*) has the canonical PAS located 7 nt downstream of the first of two stop codons arranged in tandem and the poly(A) tail is added 17 nt downstream of the “AAUAAA”; while in the late gene *lef-8* (AcMNPV’s *ORF50*), the PAS is located at 130 nt downstream of the two tandem termination codons [117]. In turn, the BmNPV’s *ORF60* late expression gene (AcMNPV’s ORF74) has two “AAUAAA” signals located at 207 and 275 nt downstream of a “TAA” stop codon [118].

However, it was not possible to explain initially how polyadenylation occurred once the transcription of the baculoviral pre-mRNA ends. One possibility postulated that the processing reaction was similar to that of other viral families such as *Poxviridae* (Smallpox), which require their own viral PAP and is independent of their transcription machinery [115]. Nevertheless, unlike other vRNAPs, there is no evidence that baculoviruses express subunits responsible for the activity of adding the poly(A) and does not have a CTD, which allows the assembly of the 3′ end processing machinery [111]. For this reason, although the baculoviral RNAP can transcribe mRNA from late genes, it was conjectured that the polyadenylation event could be mediated either by the cellular processing machinery or by other viral enzymes. In support of the last assumption, analysis of late baculoviral transcripts using 3′ RACE and sequencing have demonstrated the presence of poly(A) tail after U-rich motifs [119], which allowed to conclude that the processing of these late genes were mediated by the baculoviral RNAP through a mechanism that differed from that found in eukaryotes. According to this hypothesis, the formation of 3′ ends of baculoviral mature mRNAs could be the product of a transcription termination process after the U-rich region, followed by polyadenylation, and not of cleavage and then polyadenylation as occurs in eukaryotic transcripts [119]. This suggested that the baculoviral RNAP would have both an intrinsic transcription termination activity and a template-free nucleotide synthesis function. However, this assumption was not experimentally determined.

### 5.2. Bioinformatic Analyses

In 2003, a study conducted by our group allowed us to propose an initial model that attempted to explain the mechanism of 3′ end processing of baculoviral pre-mRNAs. This work, validated by in vitro techniques and bioinformatic algorithms, was based on the analysis of post-transcriptional regulatory regions of the gp64 locus of only eight alphabaculoviruses [120]. Likewise, it allowed us founding the most frequently sequences and positions of the conserved putative elements (USE, PAS, CS and DSE) involved in the 3′ end processing. GP64 is a transmembrane protein expressed in both early and late infection stages and the 3′-UTR contains canonical PASes located: 8 and 44 nt downstream of the stop codon in AcMNPV, AnfaMNPV (*Anagrapha falcifera MNPV*), AgMNPV (*Anticarsia gemmatalis MNPV*), BmNPV and CfMNPV (*Choristoneura fumiferana MNPV*); 64 nt downstream of the stop codon in HycuNPV (*Hyphantria cunea NPV*); 175 and 192 nt *downstream* of the stop codon in EppoNPV (*Epiphyas postvittana NPV*); and 136, 154 and 169 nt *downstream* of the stop codon in OpMNPV (*Orgyia pseudotsugata MNPV*). A deep analysis of this initial model showed that the positions of the main sequence elements involved in the polyadenylation event (USE, PAS and DSE) and the distance between them varies little from the positions determined for these elements in eukaryotic genes, unlike the position of the Aux-DSE (which has only 17% similarity to that detected in eukaryotes). Following the 5′ to 3′ orientation of the genes, the USE, which most frequent sequence is “UUUU” and its function is to promote the recruitment of processing factors, is the first signal located 5–30 nt upstream of PAS element. Between them is the “UGUA” motif, recognized by the cellular CFIm. Then, there is the PAS or PE (which most frequent sequence is “AAUAAA”), where the CPSF joins are located between 0–50 nt downstream to the stop codon and 12–42 nt upstream to the cleavage site. The CS is 4–40 nt upstream from the DSE, which is the binding site for the CstF, and has two main motifs: “UUUUU” and “GUUGU”. Finally, the Aux-DSE is located downstream of the DSE and has a G-rich motif. This sequence element interacts with regulatory factors that increase the 3′ end formation of the baculoviral pre-mRNA. Particularly, the proposed initial model agreed with some previous published information; and allowed us to detect a specific characteristic absent in eukaryotic genes, the presence of pairs of complementary sequences of 4–6 nt. One of them is located upstream of the CS and overlaps with the PAS, and the other is located downstream of the CS, overlapping the DSE. The presence of these complementary sequences could promote the formation of stem loop structures having the CS in the single stranded region.

In addition, the proposal of this initial model is consistent with the experimental data from work of Chen and collaborators [113]. Through the analysis of the AcMNPV transcriptome obtained by RNA-Seq 120 PASes were mapped, which average distance to the stop codon was 338 nt, and 77% was on average between 18–22 nt upstream of the CS. Moreover, in 58 mRNAs a U-rich region located at 2–10 nt downstream of the CS was detected. However, no transcription termination and polyadenylation events subsequent to the U-rich regions were evidenced in the transcriptome, and it was determined that only 13% of the late genes contained these upstream motifs of the detected PAS [113]. Of the total number of transcripts analyzed, 84 had a single PAS, while only in 14 multiple PASes were found. There were also 16 genomic regions that contained multiple overlapping mRNAs, which started in different positions but ended in the same PAS, a characteristic found in some viral genomes [113]. Besides, the experiment showed that the PAS was located in AU-rich genomic regions with respect to the rest of the AcMNPV genome (59%), since its AU content was 80% in the 40 nt adjacent to the PAS. Thus, the results obtained on the position of the PAS in the AcMNPV transcripts, when comparing the distances with respect to the motifs determined in eukaryotic mRNA (mammals and insects), would confirm that the baculoviral genes are processed by the cellular polyadenylation machinery from the “AAUAAA” consensus motif.

**Figure 4:**
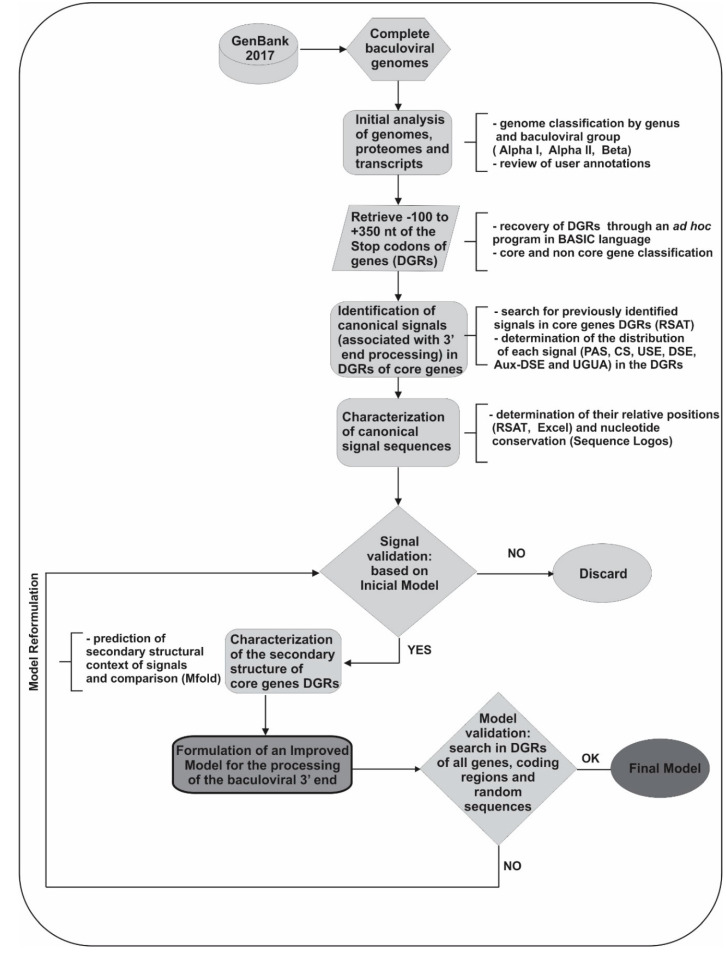
Workflow summary. A workflow diagram summarizing the bioinformatic analysis performed on downstream gene regions (DGRs: −100 to +350 relative to stop codon) of all predicted baculoviral genes is shown. 180 complete genomes (GenBank) were used, of which 53 were alphabaculoviruses Group I, 90 alphabaculoviruses Group II and 37 betabaculoviruses. The programs used in each step are indicated in parentheses. Alpha I: alphabaculoviruses Group I; Alpha II: alphabaculoviruses Group II; Beta: betabaculoviruses. PAS: Polyadenylation signal; USE: Upstream sequence element; DSE: Downstream sequence element; CS: Cleavage site: Aux-DSE: Auxiliary downstream sequence element; UGUA: motif that is eventually found upstream to the PAS and transcription end site.

**Figure 5:**
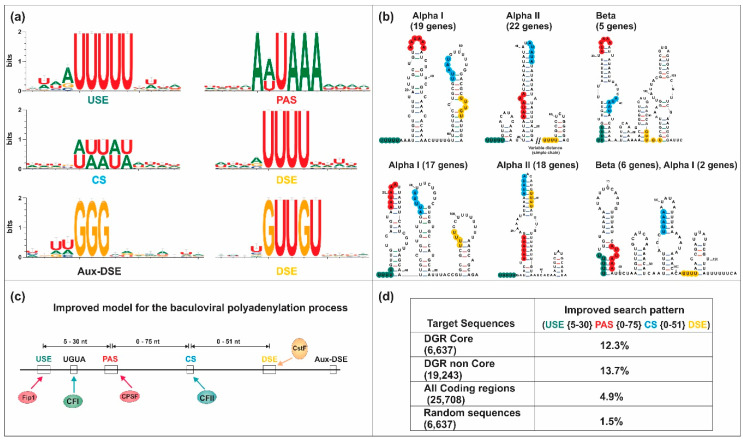
Comprehensive bioinformatic analysis of the 3′ end of baculoviral genes. (**a**) Sequence logos showing the nucleotide context of the main polyadenylation signals identified at DGR core genes. Each logo contains 237 sequences that correspond to DGR core genes in which the 6 motifs were detected (98 sequences from alphabaculoviruses Group I, 99 from alphabaculoviruses Group II and 37 from betabaculoviruses). PAS: Polyadenylation signal; USE: Upstream sequence element; DSE: Downstream sequence element; CS: Cleavage site: Aux-DSE: Auxiliary downstream sequence element. (**b**) Structural context of the polyadenylation signals. The secondary structure of the same sequences mentioned in (**a**) was determined and after a comparison, several conserved structures were detected; the 6 structures shown are the conserved ones adopted by most of the sequences used for the analysis. The number of genes in which the structures were identified is indicated in brackets. Alpha I: Alphabaculovirus Group I; Alpha II: Alphabaculovirus Group II; Beta: Betabaculovirus. (**c**) Sequence elements involved in the polyadenylation mechanism in baculoviral genes. The positions of the elements involved and the ranges of distances between them are indicated, according to the improve model proposed in our working group. The different cellular factors that would be involved are also indicated. UGUA: motif that is eventually found upstream to the PAS and transcription end site; CPSF: Cleavage and specific polyadenylation factor; CstF: Cleavage stimulation factor; CFI and CFII: Cleavage factors I and II; Fip1: Pre-mRNA 3′ end-processing factor. (**d**) Detection of the improved model postulated in different data sets for validation. Searches were carried out on the DGR of core (DGR core) and non-core (DGR non core) genes, in addition to random sequences and all coding regions (nucleotides between the initial and stop codon) of all genes of genomes used. The amount of sequences in each data set is shown in brackets. The same colors are used in all panels: USE, green; PAS, red; CS: blue; DSE: yellow.

Since there are many complete genomes, we found very interesting to verify the applicability of the proposed initial model (based on only eight sequences) in the 3′-UTR regions of all complete genomes deposited in the GenBank. Our intention to evaluate the presence of nucleotide signals similar to eukaryotes in baculoviruses is linked to the fact that, currently, protein homologous to those involved in eukaryotic polyadenylation are present in insects infected by baculoviruses, but baculovirus homologues have not yet been detected for all proteins. In this sense, the occurrence of remote baculoviral homologues is only known for two of the proteins involved in the eukaryotic polyadenylation process: RPB1 (LEF-9) and RPB2 (LEF-8) [121,122]. The structural and amino acid similarities of LEF-9 and LEF-8 with their homologues are described in detail in the work of Ruprich-Robert and Thuriaux [123]. However, it should be mentioned that the similarity is limited to very few residues that correspond to the minimal region of the active site. Considering the limited knowledge on this subject, next comprehensive structural bioinformatic analysis is required because could allow to identify some other remote baculoviral homologs linked to the polyadenylation factors, which are not yet reported. For these analyses, we started by conducting several bioinformatic approaches (MSA, pattern-searching and sequence logos) to deepen and evaluate the initial baculoviral 3′ end processing model. Briefly, 180 complete baculoviral genomes [GenBank: *Alphabaculovirus* Group l (53), *Alphabaculovirus* Group II (90) and *Betabaculovirus* (37)] were used to recover their Downstream gene regions (DGRs: −100 to +350 relative to stop codon) of all core genes (Figure 4, Supplementary Materials Table S1). To identify the signals involved in polyadenylation, pattern recognition was carried out using RSAT server [88]. Their distribution was characterized by determining the hot spots for the appearance of each motif, using filters in Microsoft Excel 2010 datasheets and ad hoc scripts. These motifs were grouped according to whether they could be functional or not (according to previous knowledge), and their relative position was characterized by determining the distances between the sequence elements present at the 3′ end of genes. Initially, and considering that there could be differences between the two classes of viral genes (early and late), we classified the DGRs of the core genes according to that classification to determine if these sequences presented differential patterns. Nonetheless, in our searches no differences were detected. After a careful analysis of MSA, the most conserved residues of each motif and its adjacent areas were determined (Figure 5a). We identified that the main cis elements were positioned in regions of the 3′ end near to the stop codon and with great “AU” content. The results also supported the ones previously obtained by our group and by Chen and collaborators: the USE was located 5–30 nt upstream of the PAS, which was located between 12 nt upstream and 7 nt downstream of the stop codon, 0–75 nt upstream of the CS and immediately downstream or overlayed with the “UGUA” motif (stand after U-rich regions); the DSE was located 21–29 nt downstream of the PAS, 0–50 nt downstream of the CS and upstream of the Aux-DSE (stand in G-rich regions). Regarding to the importance of the pre-mRNA’s structure in the baculoviral polyadenylation process, the secondary RNA structure of a subset of DGRs (87) was predicted using Mfold server, determining and analyzing consensus structures (Figure 5b). We found that the upstream region of the CS (where the PAS was positioned) was quite structured forming stems, terminal loops, or interior loops structures whereas the region where the CS was located could be less or more structured, with the CS in the loop or stem region of the terminal loops, respectively. The existence of partial or total complementarity of the cis-acting elements USE-PAS, USE-CS, PAS-CS, PAS-DSE and CS-DSE was also identified. Based on the analysis of these preliminary results, the model was modified, and the searches were repeated until an improved model was obtained (Figure 5c). Despite this, the improved model could not be detected in all 3′-UTRs of the complete genomes studied (DGRs of core and non-core genes, Figure 5d). This may be due to the fact that the model must be further refined to include all genes, although it cannot be ruled out that some sets of genes respond to different models, or that some of these predicted genes are not functional.

## 6. Conclusions

The downstream regulatory regions of the baculoviral protein genes are processed as one of the maturation steps of pre-mRNAs prior and necessary for their translation. Likewise, polyadenylation constitutes one of the many mechanisms of regulation of post-transcriptional gene expression, since it allows the appearance of different possible phenotypes from the same genotype in response to extra and intracellular stimuli. In turn, it is associated with different important biological mechanisms: the termination of transcription; the intracellular transport of the pre-mRNA to the cytoplasm; the stability and determination of its half-life; the limiting the last intron to cleave during splicing; among others. Briefly, polyadenylation involves three main steps: recognition of the PAS by the CPSF, cleavage at the CS by the formed cleavage complex, and addition of a generated poly(A) tail at the 3′ end by the PAP. In addition, it has been determined that the availability of the proteins that integrate the processing machinery and the correct positioning of the cis-acting elements, depending on their nucleotide and structural environment, constitute the two main factors that regulate the development of this biological process.

Several previous researches have attempted to characterize how the processing of the 3′ end of baculovirus genes occur and most of them agree that the polyadenylation process could have similarities with what occurs in eukaryotes. More specifically, these studies highlight that the nucleotide signals involved in this mechanism are relatively conserved in nucleotide composition and distances. Consistent with our collective findings, this mechanism would seem to be mediated exclusively by the processing machinery of the 3′ end of the host or by a combination of viral and host factors, regardless of the temporality of pre-mRNAs appearance during the infectious cycle. In other words, despite the differential transcription of genes (RNAP II and viral RNAP for early and late genes, respectively), the 3′ end of them would be processed by the host polyadenylosome complex in the infected cell nucleus, without intervention of viral proteins. However, we must also consider that there are some points that require to be analyzed to provide a better understanding. For example, it will be need to perform analyzes that involve a “de novo discovery pattern” strategy since the functional signals could be sufficiently different to be detected using patterns only based on eukaryotic events; or, besides, it will be useful to perform structural studies to finally propose a more complete functional model of the baculoviral polyadenylosome complex.

The current challenge is to continue researching in genomics through several bioinformatic approaches, in order to propose a more complete model of sequence elements involved in 3′ end processing of baculoviral transcripts, which can be applied with high specificity and effectiveness to identify 3′ ends of baculoviral protein genes, belonging to novel genomes and/or to partial sequences of this viral family. Furthermore, the information obtained will provide relevant knowledge that may be useful in making decisions for genome editions or for the synthesis of baculoviral genomes; all of them aimed to improve their applications and identifying new potential fields of action for baculoviruses.

## Figures and Tables

**Table 1:** Bioinformatics tools for PASes detection and characterization.

	Bioinformatic Tool	Description	URL	Reference
**Databases**	*EST databases*	contain sets of short cDNA sequences (500–800 nt) representing fragments of expressed genes from wide-diverse transcriptomes; used for transcripts identification and gene sequence determination	https://www.ncbi.nlm.nih.gov/genbank/dbest/	[61]
	*PolyASite*	portal to curated sets of human, mouse, and worm poly(A) sites, based on all 3′ end sequencing datasets available in the SRA nucleotide database (June 2019)	https://polyasite.unibas.ch/	[68]
	*PolyA_DB3*	contains poly(A) sites identified in several vertebrate species	https://exon.apps.wistar.org/PolyA_DB/	[69]
	*APADB*	database for mammalian APA determined by 3′ end sequencing	http://tools.genxpro.net/apadb/	[70]
	*APASdb*	database of APA sites designed to visualize the precise map and usage quantification of different APA isoforms on a genome-wide scale for all genes	http://genome.bucm.edu.cn/utr/	[71]
	*GRSDB - The ‘G’-Rich Sequences Database*	contains information on composition and distribution of putative quadruplex forming ‘G’-Rich Sequences (QGRS) in the alternatively processed (alternatively spliced or alternatively polyadenylated) mammalian pre-mRNA sequences	https://bioinformatics.ramapo.edu/grsdb/index.php	[72]
**Alignments**	*Clustal*	software for multiple sequence alignment which algorithm proceeds in a three-steps-routine, including pairwise alignment, distance matrix determination and guide tree creation to align the query sequences depending on their similarity	http://www.clustal.org/	[64]
	*Muscle*	software for MSA which algorithm is based on a three-stages-routine, consisting in a draft multiple alignment creation, its re-estimation using the Kimura distance algorithm producing a superior draft alignment, and a final refinement stage	http://www.drive5.com/muscle/	[65]
	*HMMER*	fast and sensitive homology searches using profile hidden Markov Models	http://hmmer.org/ https://www.ebi.ac.uk/Tools/hmmer/	[73]
**Pattern search**	*MEME SUITE*	online server for sequence motifs discovery and analysis	http://meme-suite.org/	[74]
	*RSAT*	analysis tools for cis-regulatory elements in genome sequences	http://rsat.sb-roscoff.fr/	[75]
	*Sequence Logos*	graphical representation of the sequence conservation in biological sequences (DNA, RNA, and proteins) created from an MSA	https://weblogo.berkeley.edu/ http://weblogo.threeplusone.com/	[76]
**Structure determination**	*Mfold*	web server for nucleic acid folding and hybridization prediction	http://unafold.rna.albany.edu/?q=mfold/RNA-Folding-Form	[77]
	*RNAstructure*	web server for RNA secondary structure prediction	http://rna.urmc.rochester.edu/RNAstructureWeb/	[78]
	*ViennaRNA Web Services*	provide programs, web services and databases related to RNA secondary structures	http://rna.tbi.univie.ac.at/	[79]
**PASes prediction**	*POLYAR*	software for PASes prediction in human sequences, based on PAS and CS functional characterization and their distance determination	http://www.mybiosoftware.com/polyar-human-polyadenylation-site-prediction.html	[80]
	*PAC*	recognition model for PASes prediction in plant sequences with a modular design and adaptable to other species	http://www.polya.org/	[81]
	*PolyA-iEP*	data mining method for PASes prediction in *A. Thaliana*, determining emerging patterns and used for descriptive and predictive analysis	http://mlkd.csd.auth.gr/PolyA/index.html	[82]
	*PolyA_SVM*	program for poly(A) sites prediction in DNA/RNA sequences and/or determines the occurrence of cis-elements	https://exon.apps.wistar.org/polya_svm/	[83]
	*Omni-PolyA*	recognition model for human PASes prediction, based on the combination of machine learning and genetic algorithms	https://www.cbrc.kaust.edu.sa/omnipolya/	[84]
	*APAlyzer*	performs 3′-UTR APA, intronic APA and gene expression analysis using RNA-Seq data	https://bioconductor.org/packages/release/bioc/html/APAlyzer.html	[85]

## Supplementary Materials

The following are available online at https://www.mdpi.com/1999-4915/12/12/1395/s1, Table S1: Baculoviral genomes analyzed.
