# Supplementary material for: Advances in the Bioinformatics Knowledge of mRNA Polyadenylation in Baculovirus Genes
Source: Viruses. 2020 Dec 6;12(12):1395. doi: 10.3390/v12121395 (PMC7762203; doi:10.3390/v12121395)
Supplement: Supplementary file 1 [file viruses-12-01395-s001.pdf]

Table S1. Baculoviral genomes analyzed.

	#	Species	Strain/Isolate/Genotype	Acronym	Accession
**Alphabaculovirus Group I**	1	*Antheraea pernyi MNPV*	Isolate L2	AnpeMNPV-L2	EF207986
	2	*Antheraea pernyi NPV*	Isolate Z	AnpeNPV-Z	NC_008035
	3	*Antheraea pernyi NPV*	Strain Liaoning	AnpeNPV-Li	LC194889
	4	*Anticarsia gemmatalis MNPV*	Isolate 2D	AgMNPV	NC_008520
	5	*Anticarsia gemmatalis MNPV*	Isolate 26	AgMNPV-26	KR815455
	6	*Anticarsia gemmatalis MNPV*	Isolate 27	AgMNPV-27	KR815456
	7	*Anticarsia gemmatalis MNPV*	Isolate 28	AgMNPV-28	KR815457
	8	*Anticarsia gemmatalis MNPV*	Isolate 29	AgMNPV-29	KR815458
	9	*Anticarsia gemmatalis MNPV*	Isolate 30	AgMNPV-30	KR815459
	10	*Anticarsia gemmatalis MNPV*	Isolate 31	AgMNPV-31	KR815460
	11	*Anticarsia gemmatalis MNPV*	Isolate 32	AgMNPV-32	KR815461
	12	*Anticarsia gemmatalis MNPV*	Isolate 33	AgMNPV-33	KR815462
	13	*Anticarsia gemmatalis MNPV*	Isolate 34	AgMNPV-34	KR815463
	14	*Anticarsia gemmatalis MNPV*	Isolate 35	AgMNPV-35	KR815464
	15	*Anticarsia gemmatalis MNPV*	Isolate 36	AgMNPV-36	KR815465
	16	*Anticarsia gemmatalis MNPV*	Isolate 37	AgMNPV-37	NC_031761
	17	*Anticarsia gemmatalis MNPV*	Isolate 38	AgMNPV-38	KR815467
	18	*Anticarsia gemmatalis MNPV*	Isolate 39	AgMNPV-39	KR815468
	19	*Anticarsia gemmatalis MNPV*	Isolate 40	AgMNPV-40	KR815469
	20	*Anticarsia gemmatalis MNPV*	Isolate 42	AgMNPV-42	KR815470
	21	*Anticarsia gemmatalis MNPV*	Isolate 43	AgMNPV-43	KR815471
	22	*Autographa californica MNPV*	Clone C6	AcMNPV	NC_001623
	23	*Autographa californica MNPV*	Strain E2	AcMNPV-E2	KM667940
	24	*Autographa californica MNPV*	Isolate WP10	AcMNPV-WP10	KM609482
	25	*Bombyx mandarina NPV*	Isolate S1	BomaNPV-S1	NC_012672
	26	*Bombyx mandarina NPV*	Isolate S2	BomaNPV-S2	JQ071499
	27	*Bombyx mori NPV*	Isolate T3	BmNPV	NC_001962
	28	*Bombyx mori NPV*	Strain Cubic	BmNPV-Cub	JQ991009
	29	*Bombyx mori NPV*	Strain Guangxi	BmNPV-Gua	JQ991011
	30	*Bombyx mori NPV*	Strain India	BmNPV-Ind	JQ991010
	31	*Bombyx mori NPV*	Strain Zhejiang	BmNPV-Zhe	JQ991008
	32	*Bombyx mori NPV*	Isolate C1	BmNPV-C1	KF306215
	33	*Bombyx mori NPV*	Isolate C2	BmNPV-C2	KF306216
	34	*Bombyx mori NPV*	Isolate C6	BmNPV-C6	KF306217
	35	*Bombyx mori NPV*	Strain Brazilian	BmNPV-Bra	KJ186100
	36	*Catopsilia pomona*	Isolate 416	CapoNPV	NC_030240
	37	*Choristoneura fumiferana MNPV*		CfMNPV	NC_004778
	38	*Choristoneura fumiferana Def MNPV*		CfDEFMNPV	NC_005137
	39	*Choristoneura murinana NPV*	Strain Darmstadt	ChmuNPV	NC_023177
	40	*Choristoneura occidentalis NPV*	Isolate BC1	ChocNPV	NC_021925

	41	*Choristoneura rosaceana NPV*	Isolate NB1	ChroNPV	NC_021924
	42	*Condylorrhiza vestigialis MNPV*		CoveMNPV	NC_026430
	43	*Dasychira pudibunda NPV*	Isolate ML1	DapuNPV	KP747440
	44	*Dendrolimus kikuchii NPV*	Strain YN	DekiNPV	JX193905
	45	*Epiphyas postvittana NPV*		EppoNPV	NC_003083
	46	*Hyphantria cunea NPV*		HycuNPV	NC_007767
	47	*Lonomia obliqua MNPV*	Isolate SP/2000	LoobMNPV	KP763670
	48	*Maruca vitrata NPV*		MaviNPV	NC_008725
	49	*Orgyia pseudotsugata MNPV*		OpMNPV	NC_001875
	50	*Philosamia cynthia riciniNPV*		PhcyNPV	JX404026
	51	*Plutella xylostella MNPV*	Isolate CL3	PlxyMNPV	NC_008349
	52	*Rachiplusia ou MNPV*		RoMNPV	NC_004323
	53	*Thysanoplusia orichalcea NPV*	Isolate P2	ThorNPV-P2	NC_019945
**Alphabaculovirus Group II**	54	*Adoxophyes honmai NPV*		AdhoNPV	NC_004690
	55	*Adoxophyes orana NPV*		AdorNPV	NC_011423
	56	*Agrotis ipsilon MNPV*		AgipMNPV	NC_011345
	57	*Agrotis segetum NPV*		AgseNPV	NC_007921
	58	*Agrotis segetum NPV*	B Isolate English	AgseNPV-Beng	NC_025960
	59	*Apocheima cinerarium NPV*		ApciNPV	NC_018504
	60	*Buzura suppressaria NPV*	Isolate Hubei	BusuNPV	NC_023442
	61	*Buzura suppressaria NPV*	Isolate Guangxi	BusuNPV-G	KM986882
	62	*Chrysodeixis chalcites NPV*		ChchNPV	NC_007151
	63	*Chrysodeixis chalcites NPV*	genotype ChchSNPV-TF1-C	ChchNPV-TF1C	JX560539
	64	*Chrysodeixis chalcites NPV*	genotype ChchSNPV-TF1-H	ChchNPV-TF1H	JX560542
	65	*Chrysodeixis chalcites NPV*	genotype ChchSNPV-TF1-B	ChchSNPV-TF1B	JX560540
	66	*Chrysodeixis chalcites NPV*	genotype ChchSNPV-TF1-G	ChchSNPV-TF1G	JX560541
	67	*Chrysodeixis chalcites NPV*	genotype ChchSNPV-TF1-A	ChchSNPV-TF1A	JX535500
	68	*Clanis bilineata NPV*	Isolate DZ1	ClbiNPV	NC_008293
	69	*Ectropis obliqua NPV*	Strain A1	EcobNPV	NC_008586
	70	*Ectropis obliqua NPV*	Strain Unioasis 1	EcobNPV-U1	KC960018
	71	*Euproctis pseudoconspersa NPV*		EupsNPV	NC_012639
	72	*Helicoverpa armigera MNPV*		HaMNPV	NC_011615
	73	*Helicoverpa armigera NPV*	Isolate Australia	HaNPV-Aus	JN584482
	74	*Helicoverpa armigera NPV*	Strain C1	HaNPV-C1	NC_003094
	75	*Helicoverpa armigera NPV*	Strain G4	HaNPV-G4	NC_002654
	76	*Helicoverpa armigera SNPV*	Strain NNg1	HaSNPV	NC_011354
	77	*Helicoverpa armigera SNPV*	Isolate H25EA1	HaSNPV-H25EA1	KJ922128

	78	*Helicoverpa armigera SNPV*	Strain AC53	HaSNPV-AC53	NC_024688
	79	*Helicoverpa armigera NPV*	Strain SP1A	HaNPV-SP1A	KJ701032
	80	*Helicoverpa armigera NPV*	Strain SP1B	HaNPV-SP1B	KJ701033
	81	*Helicoverpa armigera NPV*	Strain L1	HaNPV-L1	KT013224
	82	*Helicoverpa armigera NPV*	Strain LB1	HaNPV-LB1	KJ701029
	83	*Helicoverpa armigera NPV*	Strain LB3	HaNPV-LB3	KJ701030
	84	*Helicoverpa armigera NPV*	Strain LB6	HaNPV-LB6	KJ701031
	85	*Helicoverpa armigera NPV*	Strain AC53T4.1	HaSNPV-T4-1	KU738902
	86	*Helicoverpa armigera NPV*	Strain AC53T4.2	HaSNPV-T4-2	KU738903
	87	*Helicoverpa armigera NPV*	Strain AC53C1	HaSNPV-C1	KU738896
	88	*Helicoverpa armigera NPV*	Strain AC53C3	HaSNPV-C3	KU738897
	89	*Helicoverpa armigera NPV*	Strain AC53C5	HaSNPV-C5	KU738898
	90	*Helicoverpa armigera NPV*	Strain AC53C6	HaSNPV-C6	KU738899
	91	*Helicoverpa armigera NPV*	Strain AC53C9	HaSNPV-C9	KU738900
	92	*Helicoverpa zea NPV*		HezeNPV	NC_003349
	93	*Helicoverpa zea SNPV*	Strain HS-18	HezeSNPV-HS18	KJ004000
	94	*Helicoverpa zea SNPV*	Isolate Br/South	HezeSNPV-Br/South	KM596835
	95	*Hemileuca sp NPV*		HespNPV	NC_021923
	96	*Lambdina fiscellaria NPV*	Isolate GR15	LafiNPV-GR15	NC_026922
	97	*Leucania separata NPV*	Strain AH1	LeseNPV	NC_008348
	98	*Lymantria dispar MNPV*		LdMNPV	NC_001973
	99	*Lymantria dispar MNPV*	Isolate LdMNPV-27	LdMNPV-27	KP027546
	100	*Lymantria dispar MNPV*	Strain 3029	LdMNPV-3029	KM386655
	101	*Lymantria dispar MNPV*	Isolate 2161	LdMNPV-2161	KF695050
	102	*Lymantria dispar MNPV*	Isolate BNP	LdMNPV-BNP	KU377538
	103	*Lymantria dispar MNPV*	Isolate RR01	LdMNPV-RR01	KX618634
	104	*Lymantria dispar MNPV*	Isolate Aba624	LdMNPV-Aba624	KT626572
	105	*Lymantria dispar MNPV*	Strain 3041	LdMNPV-3041	KT626571
	106	*Lymantria dispar MNPV*	Strain 3054	LdMNPV-3054	KT626570
	107	*Lymantria dispar MNPV*	Isolate 45/0	LdMNPV-45/0	KU862282
	108	*Lymantria xylina MNPV*	Isolate LyxyMNPV-5	LyxyMNPV	NC_013953
	109	*Mamestra brassicae MNPV*	Isolate Chb1	MabrMNPV-Chb1	JX138237

	110	*Mamestra brassicae MNPV*	Isolate CTa	MabrMNPV-CTa	KJ871680
	111	*Mamestra brassicae MNPV*	Isolate K1	MabrMNPV-K1	NC_023681
	112	*Mamestra configurata NPV*	Strain A90-2	McNPV-90-2	NC_003529
	113	*Mamestra configurata NPV*	Strain A90-4	McNPV-90-4	AF539999
	114	*Mamestra configurata NPV*	Strain B	McNPV-B	NC_004117
	115	*Orgyia leucostigma NPV*	Isolate CFS77	OrleNPV	NC_010276
	116	*Peridroma sp NPV*	Isolate GR-167	PespNPV	NC_024625
	117	*Perigonia lusca SNPV*		PeluSNPV	NC_027923
	118	*Pseudoplusia includens SNPV*	IE	PsinSNPV-IE	NC_026268
	119	*Pseudoplusia includens SNPV*	IA	PsinSNPV-IA	KU669289
	120	*Pseudoplusia includens SNPV*	IB	PsinSNPV-IB	KU669290
	121	*Pseudoplusia includens SNPV*	IC	PsinSNPV-IC	KU669291
	122	*Pseudoplusia includens SNPV*	ID	PsinSNPV-ID	KU669292
	123	*Pseudoplusia includens SNPV*	IF	PsinSNPV-IF	KU669293
	124	*Pseudoplusia includens SNPV*	IG	PsinSNPV-IG	KU669294
	125	*Spodoptera exigua MNPV*		SeMNPV	NC_002169
	126	*Spodoptera exigua MNPV*	Isolate HT-SeG25	SeMNPV-HT-SeG25	HG425347
	127	*Spodoptera exigua MNPV*	Isolate HT-SeG24	SeMNPV-HT-SeG24	HG425346
	128	*Spodoptera exigua MNPV*	Isolate VT-SeAl1	SeMNPV-VT-SeAl1	HG425343
	129	*Spodoptera exigua MNPV*	Isolate HT-SeSP2A	SeMNPV-HT-SeSP2A	HG425349
	130	*Spodoptera exigua MNPV*	Isolate HT-SeG26	SeMNPV-HT-SeG26	HG425348
	131	*Spodoptera exigua MNPV*	Isolate VT-SeAl2	SeMNPV-VT-SeAl2	HG425344
	132	*Spodoptera exigua MNPV*	Isolate VT-SeOx4	SeMNPV-VT-SeOx4	HG425345
	133	*Spodoptera frugiperda MNPV*	Isolate 3AP2	SfMNPV-3AP2	NC_009011
	134	*Spodoptera frugiperda MNPV*	Isolate Nicaraguan	SfMNPV-Nic	HM595733
	135	*Spodoptera frugiperda MNPV*	Isolate Nicaraguan DefG	SfMNPV-NicG	JF899325
	136	*Spodoptera frugiperda MNPV*	Strain 19	SfMNPV-19	EU258200
	137	*Spodoptera frugiperda MNPV*	Isolate Colombian	SfMNPV-Col	KF891883
	138	*Spodoptera littoralis NPV*	Isolate AN1956	SpliNPV-AN1956	JX454574
	139	*Spodoptera litura II MNPV*		SpltMNPV-II	NC_011616
	140	*Spodoptera litura MNPV*	Strain G2	SpltMNPV-G2	NC_003102
	141	*Sucra jujuba NPV*	Isolate 473	SujuNPV-473	KJ676450
	142	*Trichoplusia ni SNPV*		TnSNPV	NC_007383

	143	*Urbanus proteus NPV*	Isolate Southern Brazil	UrprNPV	NC_029997
**Betabaculovirus**	144	*Adoxophyes orana GV*		AdorGV	NC_005038
	145	*Adoxophyes orana GV*	Strain Miyazaki	AdorGV-Miy	KM226332
	146	*Agrotis segetum GV*		AgseGV	NC_005839
	147	*Agrotis segetum GV*	Strain L1	AgseGV-L1	KC994902
	148	*Agrotis segetum GV*	Strain DA	AgseGV-DA	KR584663
	149	*Choristoneura occidentalis GV*		ChocGV	NC_008168
	150	*Clostera anachoreta GV*	Isolate HBHN	ClanGV	NC_015398
	151	*Clostera anastomosis GV*	Strain Henan	CalGV	NC_022646
	152	*Cnapahlocrocis medinalis GV*	Strain Enping	CnmeGV-E	NC_029304
	153	*Cnapahlocrocis medinalis GV*		CnmeGV	KP658210
	154	*Cryptophlebia leucotreta GV*		CrleGV	NC_005068
	155	*Cydia pomonella GV*		CpGV	NC_002816
	156	*Cydia pomonella GV*	Isolate CpGV-S	CpGV-S	KM217573
	157	*Cydia pomonella GV*	Isolate CpGV-I12	CpGV-I12	KM217576
	158	*Cydia pomonella GV*	Isolate CpGV-M	CpGV-M	KM217575
	159	*Cydia pomonella GV*	Isolate CpGV-I07	CpGV-I07	KM217574
	160	*Diatraea saccharalis GV*	Isolate DisaGV-Parana-2009	DisaGV-P09	NC_028491
	161	*Epinotia aporema GV*		EpapGV	NC_018875
	162	*Helicoverpa armigera GV*		HearGV	NC_010240
	163	*Mocis sp GV*	Isolate Southern Brazil	MospGV	NC_029996
	164	*Mythimna unipuncta GV*	Isolate MyunGV#8	MyunGV	NC_033780
	165	*Phthorimaea operculella GV*		PhopGV	NC_004062
	166	*Phthorimaea operculella GV*	Isolate SA	PhopGV-SA	KU666536
	167	*Pseudaletia unipuncta GV*	Strain Hawaiin	PsunGV	NC_013772
	168	*Pieris rapae GV*	Strain E3	PiraGV-E3	GU111736
	169	*Pieris rapae GV*	South Korea	PiraGV-SK	JX968491
	170	*Plodia interpunctella GV*	Isolate Cambridge	PlinGV	NC_032255
	171	*Plutella xylostella GV*		PxGV	NC_002593
	172	*Plutella xylostella GV*	Isolate SA	PlxyGV-SA	KU666537
	173	*Plutella xylostella GV*	Isolate C	PlxyGV-C	KU529791
	174	*Plutella xylostella GV*	Isolate K	PlxyGV-K	KU529792
	175	*Plutella xylostella GV*	Isolate M	PlxyGV-M	KU529793
	176	*Plutella xylostella GV*	Isolate T	PlxyGV-T	KU529794
	177	*Spodoptera frugiperda GV*	Isolate VG008	SpfrGV	NC_026511
	178	*Spodoptera litura GV*	Strain K1	SpltGV	NC_009503
	179	*Trichplusia ni GV*	Isolate LBIV-12	TrniGV-LBIV12	KU752557
	180	*Xestia c nigrum GV*		XecnGV	NC_002331
